# Soluble CD59 in peritoneal dialysis: a potential biomarker for peritoneal membrane function

**DOI:** 10.1007/s40620-020-00934-7

**Published:** 2020-12-11

**Authors:** Bernardo Faria, Mariana Gaya da Costa, Carla Lima, Loek Willems, Ricardo Brandwijk, Stefan P. Berger, Mohamed R. Daha, Manuel Pestana, Marc A. Seelen, Felix Poppelaars

**Affiliations:** 1 Department of Internal Medicine, Division of Nephrology, University of Groningen, University Medical Center Groningen, Groningen, The Netherlands; 2 Nephrology and Infectious Disease R&D Group, INEB, Institute of Investigation and Innovation in Health (i3S), University of Porto, Al. Professor Hernâni Monteiro, 4200-319 Porto, Portugal; 3 Hospital São Teotônio, Viseu, Portugal; 4 Hycult Biotech, Uden, The Netherlands; 5 Department of Nephrology, University of Leiden, Leiden University Medical Center, Leiden, The Netherlands

**Keywords:** Complement, Dialysis, Chronic kidney disease, Innate immunity

## Abstract

**Introduction:**

Various studies have reported the importance of complement regulators in preventing mesothelial damage during peritoneal dialysis (PD). Its assessment, however, is limited in clinical practice due to the lack of easy access to the peritoneal membrane. Recently, a soluble form of the complement regulatory protein CD59 (sCD59) has been described. We therefore aimed to investigate the role of sCD59 in PD.

**Methods:**

Plasma sCD59 was measured in 48 PD patients, 41 hemodialysis patients, 15 non-dialysis patients with chronic kidney disease and 14 healthy controls by ELISA (Hycult; HK374-02). Additionally, sCD59 and sC5b-9 were assessed in the peritoneal dialysate.

**Results:**

sCD59 and sC5b-9 were detectable in the peritoneal dialysate of all patients, and marginally correlated (*r* = 0.27*, P* = 0.06). Plasma sCD59 levels were significantly higher in PD patients than in patients with chronic kidney disease and healthy controls, but did not differ from hemodialysis patients. During follow-up, 19% of PD patients developed peritoneal membrane failure and 27% of PD patients developed loss of residual renal function. In adjusted models, increased sCD59 levels in the dialysate (HR 3.44, 95% CI 1.04–11.40, *P* = 0.04) and in plasma (HR 1.08, 95% CI 1.01–1.17, *P* = 0.04) were independently associated with the occurrence of peritoneal membrane failure. Higher plasma levels of sCD59 were also associated with loss of residual renal function (HR 1.10, 95% CI 1.04–1.17, *P* < 0.001).

**Conclusions:**

Our study suggests that sCD59 has potential as a biomarker to predict peritoneal membrane function and loss of residual renal function in PD, thereby offering a tool to improve patient management.

**Graphic abstract:**

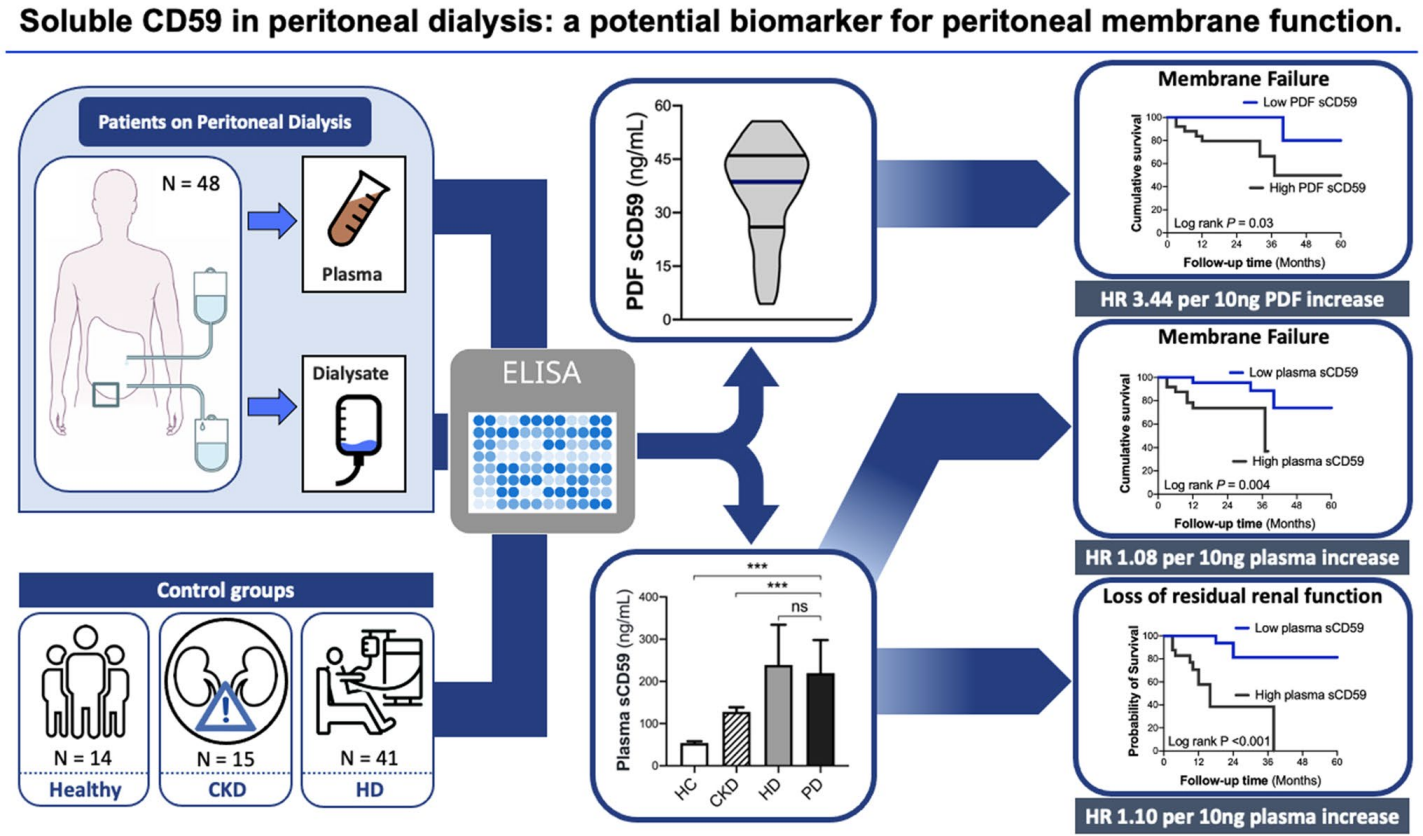

**Supplementary Information:**

The online version contains supplementary material available at 10.1007/s40620-020-00934-7.

## Introduction

Peritoneal dialysis (PD) remains an under-used dialysis technique despite being a cost-effective alternative, offering several advantages over hemodialysis (HD) [1]. In PD, there is an urgent need for biomarkers that identify patients who are at risk for complications and to guide personalized interventions that can improve their clinical outcome [2]. Preservation of residual renal function (RRF) is of paramount importance in dialysis patients [3], while maintaining peritoneal membrane integrity is key for the long-term success of PD [4].

Chronic inflammation in dialysis is increasingly recognized as a risk factor for morbidity and mortality in PD patients [5]. The complement system is a vital part of innate immunity. Complement can be activated via three pathways, all leading to the generation of the membrane attack complex (C5b-9). Tight regulation of this system by complement regulatory proteins (Cregs) prevents unwanted complement activation and subsequent inflammation and tissue injury [6]. The mesothelial cells of the human peritoneum are known to express Cregs (i.e. CD46, CD55 and CD59) [7, 8]. Animal models of PD have demonstrated that impairment of Cregs, especially CD59, results in uncontrolled local complement activation triggering severe inflammation and progressive peritoneal injury [9, 10]. In PD patients, expression of the Cregs is reduced on mesothelial cells due to the therapy itself [11]. Sei et al. [8] showed that modified expression of Cregs on the peritoneum is associated with peritoneal membrane function in PD patients. Despite these promising findings, clinical exploitation is limited due to the lack of easy access to the peritoneal membrane.

A soluble form of CD59 (sCD59) has recently been described. Previous studies have demonstrated that sCD59 is found in various body fluids and is associated with cellular damage [12–14]. CD59, also called the membrane attack complex-inhibitory protein, is an 18–21 kDa GPl-anchored protein that prevents the incorporation and polymerization of C9 on cell membranes and is the main regulator of C5b-9 [6]. We hypothesized that sCD59 is a surrogate marker for membrane function in PD patients. To test this hypothesis, we determined sCD59 in the peritoneal dialysis fluid (PDF) and plasma of PD patients. To characterize the relationship between sCD59 and local complement activation, levels of sC5b-9 were determined in the PDF. Furthermore, we also set out to identify determinants of PDF and plasma levels of sCD59 in this population. Finally, we determined the association between Cregs with peritoneal membrane failure (PMF) and loss of RRF by using the sCD59 levels in the PDF and plasma.

## Methods

### Study design

Forty-eight adult PD patients with stage 5 chronic kidney disease (CKD) were recruited from the Peritoneal Dialysis Unit at Hospital São Teotónio, Viseu, Portugal. Exclusion criteria were dialysis vintage less than 3 months, presence of active inflammation prior to the sample collection and peritonitis in the previous 3 months. Plasma EDTA samples and PDF were collected after an overnight dwell with 1.36% glucose solution. Additionally, plasma EDTA samples were collected from control groups: 41 HD patients, 15 non-dialysis CKD patients and 14 age- and sex-matched healthy controls. These patients were recruited at the Hospital de Braga, Braga, Portugal. Samples were centrifuged within 30 min of collection (3500 rpm, 15 min, − 4 °C) and stored in aliquots at − 80 °C. Prior to analysis, samples were thawed and cleared by centrifugation.

### Clinical and laboratory measurements

Clinical data was retrieved from the archives (Table 1). Body composition analysis was performed by bioimpedance spectroscopy (BCM, Fresenius Medical Care, Germany). The modified peritoneal equilibration test (PET) was used as a functional assessment of the peritoneal membrane, through ultrafiltration volume measurement and transport status defined by the dialysate-to-plasma concentration ratio (D/P) for creatinine. Protein loss was estimated from the protein concentrations at the end of the 4 h-dwell from the PET.

**Table 1:** Determinants of peritoneal membrane failure or loss of residual kidney function during follow-up

	Characteristics	Peritoneal membrane failure				Loss of residual kidney function			
		Univariate analysis		Multivariate analysis		Univariate analysis		Multivariate analysis	
	PD patients (*n* = 48)	St. Beta	*P*-value	St. Beta	*P*-value	St. Beta	*P*-value	St. Beta	*P*-value
Plasma sCD59 (ng/mL)	220 [133–298]	**1.081**	**0.001**	**1.082**	**0.040**	**1.122**	** < 0.001**	**1.104**	**0.001**
PDF sCD59 (ng/mL)	38.6 [26–46]	**2.003**	**0.035**	**3.441**	**0.043**	1.042	0.08		
D/P-ratio of sCD59	0.16 [0.11–0.21]	0.744	0.51			0.325	0.15		
PDF sC5b-9 (ng/mL)	70.5 [39–83]	1.009	0.34			0.994	0.42		
Age (years)	59 [50–67]	1.031	0.33			0.982	0.40		
Sex (Female)	18 (38)	4.440	0.16			0.668	0.47		
Dialysis vintage (months)	12 [3–33]	1.006	0.64			**1.030**	**0.007**	**1.049**	**0.003**
Residual renal function (mL/min/1.73m^2^)	5.3 [3.3–7.4]	**0.716**	**0.037**	0.869	0.48	**0.416**	** < 0.001**	1.205	0.10
Lean tissue index (kg/m^2^)	15.1 [13.5–17.2]	1.094	0.39			0.910	0.31		
Fat tissue index (kg/m^2^)	10.3 [6.6–13.8]	0.897	0.16			1.024	0.59		
Body mass index (BMI) (kg/m^2^)	27.3 [23.2–29.5]	0.965	0.34			1.027	0.51		
Overhydration (%)	6.9 [0–15.3]	**1.087**	**0.011**	**1.069**	**0.048**	1.039	0.19		
Mean arterial pressure (mm/Hg)	99.8 [87–106]	1.014	0.56			**1.053**	**0.022**	1.065	0.054
Baseline transport status (D/P creatinine)	0.70 [0.66–0.76]	518.3	0.17			2.790	0.79		
Protein loss (g/dL)	0.07 [0.05–0.08]	1.036	0.99			0.001	0.44		
Diabetes (%)	7 (15)	1.304	0.80			0.827	0.80		
Automated PD (%)	2 (4)	23.34	0.62			1.667	0.63		

Patient characteristics are described as median [IQR] or number (%) for all 48 peritoneal dialysis (PD) patients. Peritoneal membrane failure was defined as a composite outcome of either ultrafiltration failure or failure to achieve minimum small solute dialysis adequacy (Kt/v 1.7). Loss of residual renal function was defined as an average clearance of urea and creatinine lower than 2 mL/min/1.73 m^2^. Univariate Cox regression analysis of outcome with clinical parameters was conducted. Next, multivariate Cox regression using the forward selection was performed with parameters that significantly associated (*P*-value < 0.05) in univariate analysis, to identify independent determinants of outcome. Data are presented as standardized beta coefficient with corresponding *P*-value. Bold letters indicate a *P*-value < 0.05. BMI, body mass index; PD, peritoneal dialysis; PDF, peritoneal dialysis fluid; sCD59, soluble CD59; sC5b-9, soluble C5b-9; D/P, Dialysate-to-plasma concentration ratio

### Quantification of soluble CD59 and C5b-9

Levels of sCD59 were measured in samples by ELISA according to the manufacturers’ instructions (HK374-02; Hycult Biotech, Uden, The Netherlands). Levels of sC5b-9 were measured in the PDF by ELISA as previously described [15–18].

### Definition of endpoint

The primary end-point was the time to onset of PMF and loss of RRF. The secondary outcome was transport status after 12 months and loss of diuresis. PMF was defined as a composite outcome of either ultrafiltration failure or failure to achieve minimum small solute dialysis adequacy (Kt/v 1,7) [19]. Loss of RRF was defined as an average clearance of urea and creatinine (CrUCL) lower than 2 ml/min/1.73  m^2^.[20] Loss of diuresis was defined as a urinary output of less than 400 ml per day, as described elsewhere [21]. Lastly, follow-up data included transport status 12 months after sampling measured by D/P for creatinine using the modified PET. The range of the follow-up period was 57 months, namely between 3 and 60 months.

### Statistical analysis

Statistical analysis was performed using IBM SPSS 25.0 (IBM Corporation, USA). Laboratory measurements are shown as median with interquartile range (IQR). Comparisons between PD, HD, CKD patients and healthy controls were made by Kruskal–Wallis test followed by post-hoc analysis. Correlations were assessed using Spearman’s correlation coefficient (r). Univariate and multivariate logistic and Cox regression analyses were performed to determine the association between sCD59, sC5b-9, clinical baseline parameters and outcomes. Survival to PMF and loss of RRF were also assessed through Kaplan–Meier survival analysis using the log-rank test. *P*-values < 0.05 were considered to be statistically significant.

## Results

### Patient cohort

The baseline characteristics are shown in Table 1. 46 of the PD patients were on continuous ambulatory peritoneal dialysis (CAPD) and two were on automated peritoneal dialysis (APD). The median age was 59 years [50–66] and 38% of the patients were female. Fifteen percent of the patients were diabetic. Based on the transport status at baseline, 75% were classified as high-average or high transporters (D/P > 0.65), and 68% were classified as such 1 year later. During median follow-up time of 14 months [8–36], nine PD patients (19%) developed PMF, while loss of RRF occurred in 13 patients (27%).

### Local and systemic levels of sCD59

**Fig. 1:**
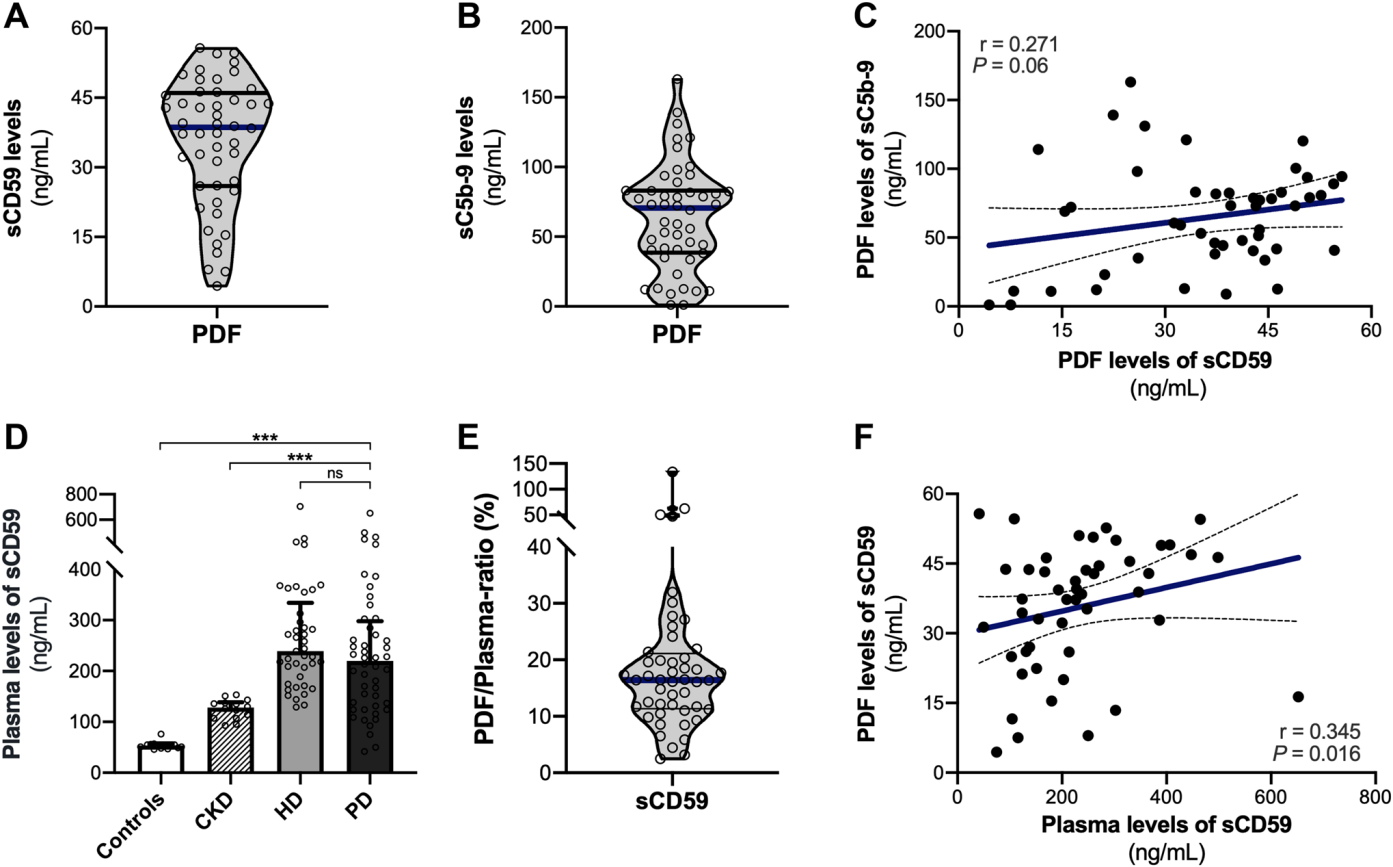
Local and systemic levels of soluble CD59 in peritoneal dialysis patients. **a** Violin plot is shown for soluble CD59 (sCD59) levels in the peritoneal dialysis fluid (PDF). The width of the shape indicates the probability density of patients with a given result. The lines represent the median (blue horizontal line), interquartile range (25th–75th percentile, black horizontal lines). **b** Violin plot is shown for soluble C5b-9 (sC5b-9) levels in the PDF. sC5b-9 was detectable in the peritoneal dialysate of all patients (n = 48). **c** The correlation of PDF levels of sCD59 with sC5b-9 using the Spearman Rank correlation coefficient. The dashed lines show the 95% confidence interval for the regression line (blue). **d** sCD59 plasma levels were determined in; healthy controls (n = 14), non-dialysis dependent chronic kidney disease (CKD) patients (n = 15), hemodialysis (HD) patients prior to dialysis (n = 41), PD patients (n = 48). Average age was 56 ± 4 years in healthy controls, 77 ± 11 years in CKD patients, and 66 ± 16 years in HD patients, and 64%, 60% and 70% were male, respectively. Data are presented as median plus interquartile range and were analyzed by Kruskal Wallis test with an option for multiple comparisons (***P < 0.001). **e** Violin plot for the PDF—plasma ratio of sCD59 in dialysis patients (n = 48). The ratio was calculated per patient by dividing the PDF level by the plasma level and multiplied by 100%. **f** The correlation of PDF levels and plasma levels of sCD59 using the Spearman Rank correlation coefficient (r represents the Spearman's rho). sCD59 was measured using an enzyme-linked immunosorbent assay (ELISA; Hycult; HK374-02)

In all patients, sCD59 was detected in the PDF and median levels were 39 ng/mL [26–46] (Fig. 1a). Median PDF levels of sC5b-9 were 70.5 ng/mL [39–83], which due its high molecular size (> 1000 kDa) indicates local complement activation in the peritoneal cavity (Fig. 1b). PDF levels of sCD59 were marginally correlated with sC5b-9 PDF levels (Fig. 1c, *r* = 0.271; *P* = 0.06). Median plasma levels of sCD59 were 220 ng/mL [133–298] in PD patients, whereas levels In HD patients, non-dialysis CKD patients and healthy controls were 239 ng/mL [182–334], 128 ng/mL [107–139], and 53 ng/mL [49–55], respectively (Fig. 1d). Systemic levels of sCD59 were significantly higher in PD patients compared to non-dialysis CKD patients and healthy controls (*P* < 0.001), but not significantly different between HD and PD patients (*P* = 0.20). These findings demonstrate the presence of sCD59 in the PDF and that systemic levels of sCD59 are increased in dialysis patients.

### Determinants of local and systemic sCD59

**Fig. 2:**
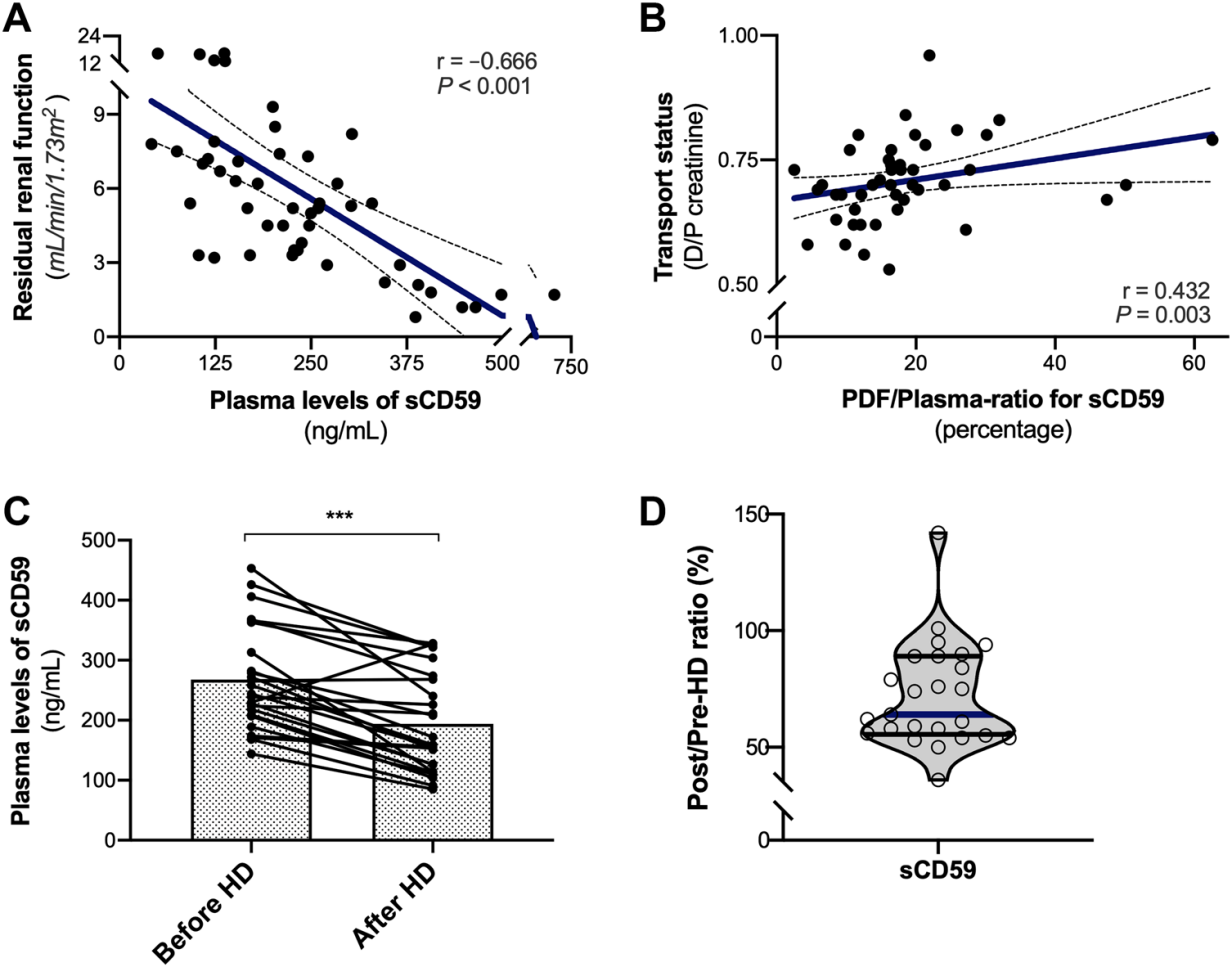
The relationship between soluble CD59 and diafiltration. **a** The correlation between residual renal function and plasma sCD59 and **b** the correlation between baseline transport status and the PDF/plasma ratio of sCD59 using the Spearman Rank correlation coefficient (r represents the Spearman's rho). The dashed lines show the 95% confidence interval for the regression line (blue). A significant correlation was found between the plasma sCD59 levels and residual renal function, and sCD59 ratio and transport status. **c** HD significantly reduced plasma sCD59 levels (***P < 0.001). **d** Violin plot for the pre-HD/post-HD ratio of plasma sCD59 levels in dialysis patients (n = 25). The ratio was calculated per patient by dividing the pre-HD level by the post-HD level and multiplied by 100%. The width of the shape indicates the probability density of patients with a given result. The lines represent the median (blue horizontal line), interquartile range (25th–75th percentile, black horizontal lines)

We next assessed the relationship between local and systemic levels of sCD59 in PD. On average, PDF levels of sCD59 were approximately 20% of those in matched plasma (Fig. 1e). Regression analyses were used to identify determinants of sCD59 levels in PD (Supplementary data). Relative overhydration was the only identified determinant of PDF sCD59 in regression analysis (Table S1, *P* = 0.014). Although plasma sCD59 weakly, but significantly, correlated with sCD59 in the PDF (Fig. 1f, *r* = 0.35; *P* = 0.016), plasma sCD59 was not significantly associated with PDF sCD59 (Table S1, *P* = 0.11). In multivariate analysis using forward selection, RRF (*P* < 0.001) and mean arterial blood pressure (*P* = 0.004) were determinants of plasma sCD59 and this model explained 49% of the variation in plasma levels (Table S2). In accordance, plasma sCD59 levels strongly correlated with RRF (Fig. 2a, *r* = –0.67; *P* < 0.001). Most important, the correlation between mean arterial pressure and plasma sCD59 levels remained after adjusting for RRF (adjusted *r* = 0.43; P = 0.004). Exclusion of the 2 APD patients did not impact our results (data not shown). In addition, no differences were found in sCD59 levels between diabetic and non-diabetic PD patients. Collectively, our findings indicate that volume overload (overhydration) is associated with higher local levels of sCD59, while increased blood pressure is associated with higher systemic levels of sCD59. The association between plasma sCD59 and RRF indicates that urinary excretion might be the main route of elimination.

### sCD59 kinetics in dialysis

We next set out to explore the kinetics of sCD59 in dialysis. We found that the PDF/plasma ratio of sCD59 significantly correlated with baseline transport status (Fig. 2b, *r* = 0.43; *P* = 0.003). Considering that plasma sCD59 levels were similar among dialysis modalities, we also determined sCD59 kinetics in HD. Further analysis of plasma sCD59 in a subgroup of 25 HD patients demonstrated a significant reduction in plasma sCD59 levels during a HD session (Fig. 2c, *P* < 0.001). Plasma sCD59 levels at the beginning were 244 ng/mL [208–338] and 159 ng/mL [120–271] at the end of the session. Systemic sCD59 decreased during dialysis in 84% of HD patients, increased in 4% and remained stable in 12% (= reduction or increase < 10%). Overall, the median reduction in sCD59 levels during HD was 36% [11–45] (Fig. 2d), which is consistent with the reduction ratios reported for factor D, another complement protein with a similar molecular size [22]. In conclusion, the findings of sCD59 in HD are consistent with dialysis kinetics of middle molecules, although absorption or binding to complement activation products on HD membrane cannot be excluded.

### sCD59 predicts PMF and loss of RRF during follow-up.

**Fig. 3:**
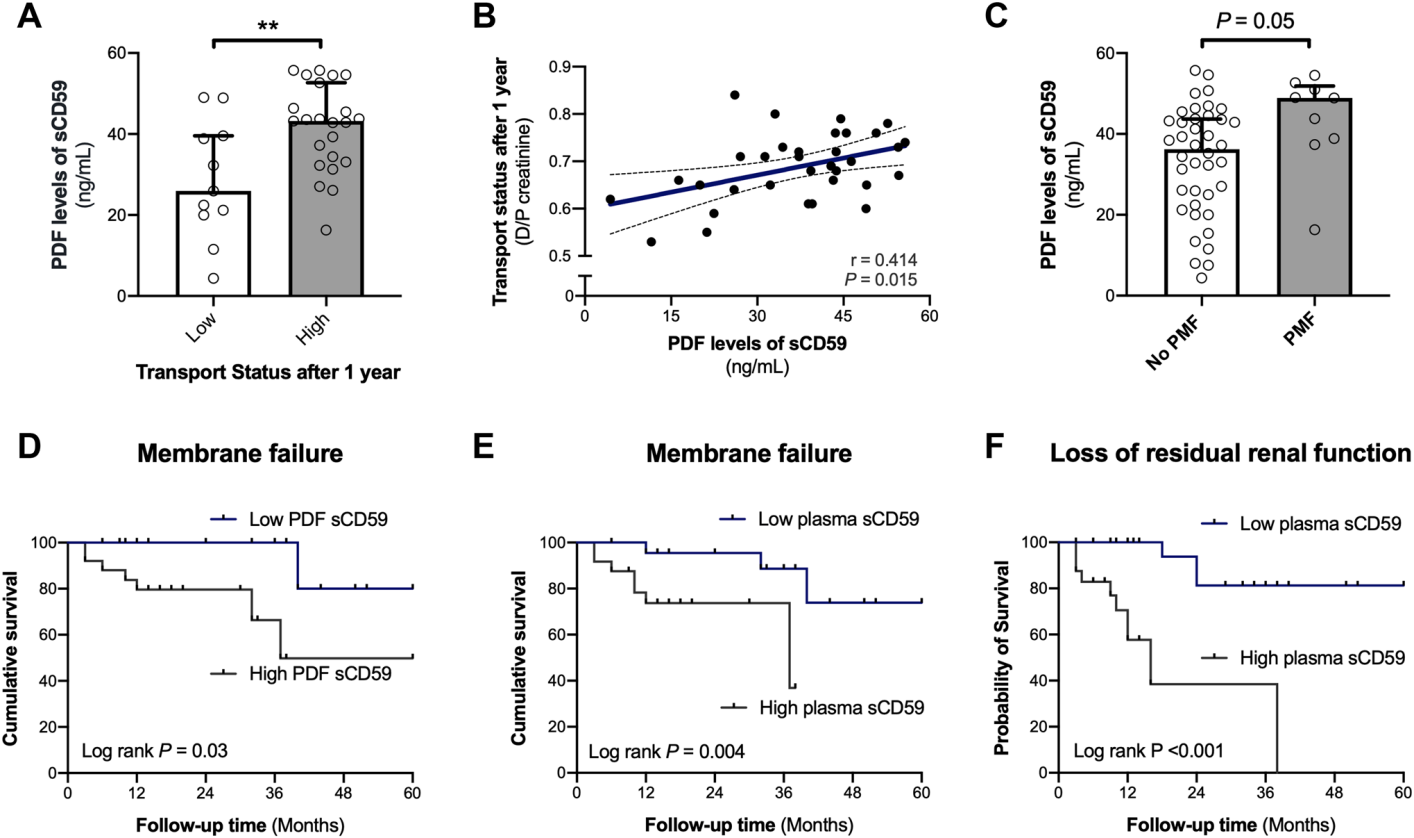
Soluble CD59 as a biomarker for transport status, membrane failure and loss of residual renal function during follow up. **a** The difference in peritoneal dialysis fluid (PDF) levels of soluble CD59 (sCD59) were analyzed between PD patient groups with low (D/P creatinine < 0.65) and high (D/P creatinine > 0.65) transport status after 1 year of follow-up. **b** The correlation between PDF sCD59 levels and transport status after 1 year using the Spearman Rank correlation coefficient (r represents the Spearman's rho). The dashed lines show the 95% confidence interval for the regression line (blue). **c** The difference in PDF sCD59 levels between PD patients with and without peritoneal membrane failure (PMF) during follow-up. Data are presented as median plus interquartile range and were analyzed by Mann–Whitney test (**P < 0.01). Cumulative event-free survival for peritoneal membrane failure among PD patients with low and high sCD59 levels in the PDF (**d**) or in the plasma (**e**). Cumulative event-free survival for loss of residual renal function among PD patients with low and high plasma levels of sCD59 levels (**f**). Log-rank test was used to compare the incidence of PMF and loss of residual renal function between the groups. High sCD59 levels in the PDF (> 38.6 ng/mL) and plasma (> 219 ng/mL) are both associated with lower survival of the peritoneal membrane. High plasma CD59 (> 219 ng/mL) was significantly associated with loss of residual renal function

We continued to investigate the association of local and systemic sCD59 levels with outcome in PD patients. According to their 1-year transport status, sCD59 PDF levels were significantly higher in the high-average/high group (Fig. 3a, 46 [43–50] versus 26 [16–34]; *P* < 0.001). PDF sCD59 levels correlated with D/P after 1 year (Fig. 3b, *r* = 0.41; *P* = 0.015). In univariate analysis, PDF sCD59 levels were associated with transport status after 1 year of follow-up. However, in multivariate analysis, PDF sCD59 levels were no longer significantly associated with transport status after 1 year (*P* = 0.08, Table S3).

For further analysis, the median was used as cut-off to divide PD patients into subgroups with high (> median) or low (< median) sCD59 levels. A trend was seen for higher sCD59 levels in the PDF of patients who subsequently developed PMF (Fig. 3c, *P* = 0.05). Kaplan–Meier curves showed a higher incidence of PMF in PD patients with high PDF sCD59 levels (> 38.6 ng/mL, Fig. 3d, *P* = 0.03) and high plasma sCD59 levels (> 219 ng/mL, Fig. 3e, *P* = 0.004). Furthermore, a higher occurrence of loss of RRF was seen in PD patients with high plasma levels of sCD59 (> 219 ng/mL, Fig. 3f, *P* < 0.001). Univariate analysis showed that baseline RRF, overhydration, PDF sCD59 (per 10 ng increase; Hazard Ratio (HR) 2.00; 95% Confidence Interval (CI), 1.05–3.82; *P* = 0.04), plasma sCD59 (per 10 ng increase; HR 1.08; 95% CI, 1.04–1.13; *P* = 0.001) were all associated with PMF during follow-up (Table 1). In the final model, using multivariate analysis with a forward selection, plasma and PDF sCD59 (as a continuous variable) and overhydration were significantly associated with PMF (Table 1). After adjustment, plasma sCD59 levels were associated with a hazard ratio of 1.08 (per 10 ng increase; 95% CI, 1.01–1.17; *P* = 0.04) and PDF CD59 levels with a hazard ratio of 3.44 (per 10 ng increase; 95% CI, 1.04–11.40; *P* = 0.04) for PMF. Furthermore, in univariable analysis, plasma sCD59 (per 10 ng increase; HR 1.12; 95% CI, 1.07–1.18; *P* < 0.001), mean arterial pressure, RRF at baseline and dialysis vintage were significantly associated with loss of RRF (Table 1). In the final model, using multivariate analysis with a forward selection, plasma sCD59 (as a continuous variable) and dialysis vintage were significantly associated with loss of RRF. After adjustment, plasma sCD59 levels were associated with a hazard ratio of 1.10 (per 10 ng increase; 95% CI, 1.04–1.17; *P* = 0.001) for loss of RRF. Accordingly, in unadjusted and adjusted models, plasma sCD59 was also significantly associated with loss of diuresis during follow-up (*P* = 0.005, Table S4).

## Discussion

CD59 is an essential regulator that prevents unwanted complement activation on host cells, as illustrated by the hemolysis seen in CD59 knockout mice and in paroxysmal nocturnal hemoglobinuria (PNH) [23, 24]. Because of its essential role in preventing damage to healthy cells, CD59 is expressed on virtually all tissues, including the inner mesothelial layer of the peritoneal membrane [7, 8]. In the current study, we report the presence and clinical relevance of a soluble form of CD59 in PD. Furthermore, our study revealed that sCD59 is partly removed by dialysis. The major findings of this study are that sCD59 is associated with PMF and progression towards loss of RRF, two highly relevant clinical outcomes in PD.

To our knowledge, our study is the first to investigate the role of sCD59 in relation to outcome in PD. An important observation in this study was higher plasma levels of sCD59 in patients with more advanced renal dysfunction. In accordance, baseline RRF was shown to be an independent determinant of plasma sCD59 levels in PD patients. The increase in plasma sCD59 could therefore be the result of reduced excretion. Previously, Lehto et al. [13] demonstrated urinary excretion of sCD59 in CKD patients and healthy controls. However, besides RRF, mean arterial pressure was another determinant of systemic sCD59. Since CD59 is highly expressed on vascular endothelial cells [25], we propose that hemodynamic stress may lead to endothelial dysfunction and subsequent sCD59 release. No differences were observed in systemic levels of sCD59 between PD and HD, indicating that the dialysis modality does not impact sCD59 levels.

The behavior of sCD59 during hemodialysis displays a kinetic profile comparable to other middle molecules. Likewise, in our PD cohort, the observed sCD59 dialysate-to-plasma is also similar to those of factor D and β2-microglobulin [26, 27]. Additional studies should be performed to test whether sCD59 can be used as a marker of dialysis adequacy. The sCD59 in the PDF could originate from the plasma through convective transport across the large pores in the peritoneal membrane, or locally through shedding from the mesothelial cells of the peritoneal membrane. However, it is difficult to disentangle the contribution of convection/clearance from local production. On the one hand, we observed that the reduction ratios and dialysate-to-plasma ratios for sCD59 found in HD and PD are consistent with convection as a removal mechanism of sCD59 in different dialysis modalities. However, in contrast, plasma sCD59 was not a significant determinant of PDF sCD59 in our multivariate analysis. These findings hint towards local release of sCD59 in the PDF rather than a systemic origin due to diffusion and convection. Overall, we speculate that the sCD59 in the PDF predominantly originates from shedding of CD59 from mesothelial cells, while clearance from plasma sCD59 potentially plays a minor role.

We cannot be definitive concerning whether sCD59 retains its complement regulatory function, although a trend towards a positive correlation between sCD59 and sC5b-9 in the PDF was found. Previously, it has been demonstrated that sCD59 can retain its regulatory activity [12], while others have reported an association between increased sCD59 levels and increased complement activation [13, 14]. Nevertheless, it is reasonable to assume that shedding of CD59 would make mesothelial cells more vulnerable to complement activation on their surface. Ideally, future studies should, therefore, investigate whether PDF levels of sCD59 coincide with complement deposition on the peritoneal membrane. Furthermore, various mechanisms have been proposed to explain the shedding of CD59 [14, 28]. Although our data do not allow conclusions regarding the mechanisms for sCD59 in the PDF, overhydration was shown to be the only independent determinant. This suggests that local shedding might be induced by hydrostatic pressure on the peritoneal membrane.

The use of sCD59 as a biomarker might be of clinical relevance for the early detection of PD patients at risk of PMF. Interestingly, plasma and PDF levels of sCD59 were both independently associated with a higher risk of PMF, whereas baseline transport status and RRF were not. These data suggest that systemic and local sCD59 reflect distinct processes that both influence membrane function in patients treated with PD. In accordance, Lambie et al. [5] demonstrated in PD patients that the local and the systemic inflammatory response are uncoupled and independent. Since mean arterial blood pressure was an independent determinant of plasma sCD59, we propose that the mechanism by which plasma sCD59 may be related to membrane function is via PD-induced vasculopathy [29]. In experimental models, complement activation has been shown to lead to inflammation and fibrosis of the peritoneal membrane [9, 29]. Furthermore, complement proteins, such as Factor B and Factor I, have been found in proteomic analysis of the PDF of PD patients who eventually progress to encapsulating peritoneal fibrosis, a deadly condition associated with a fibrotic phenotype of the peritoneal membrane [30]. Thus, alternatively, sCD59 in PDF may therefore be related to inflammation-induced peritoneal fibrosis.

Another major outcome in the current study was the loss of RRF. Plasma sCD59 levels were surprisingly a better predictor than baseline RRF. In line with our theory of CD59 shedding from endothelial cells after hemodynamic stress [14], PD-induced vasculopathy could also explain its association with future loss of RRF as a common pathway of cardiovascular disease and interplay to produce these outcomes. The potential relationship between sCD59 and PMF and RRF loss may however rely on other effector mechanisms. In fact, the role of CD59 in modulating T-cell immunity has been previously described [31]. Fittingly, T-cell immunity and inflammation have also been implicated in both PMF [32] and progressive loss of RRF [33]. The association of sCD59 with PD outcomes may therefore be related to the relationship with T-cell immunity.

This study has some limitations. First, it is a single center study with a limited number of patients. This precludes definitive conclusions regarding the predictive value of sCD59 on PD outcomes, but rather shows an association that must be further confirmed. Second, sCD59 was not compared to other established biomarkers in PD. Third, the low number of events led us to classify PMF as a composite outcome. Finally, the number of patients reaching anuria during follow-up was relatively low. In contrast, strengths include the hard and clinically relevant end points (PMF and loss of RRF) and the multiple control groups (HD, CKD and healthy controls). Costs and availability are critical aspects for considering the application of biomarkers in clinical practice. The ELISA used for the measurement of sCD59 is a commercial kit and is widely available. The expected costs per sample are currently between 15 and 20 US dollars, depending on the region where the assay is performed. More importantly, after additional validation, cost-effectiveness analyses need to be performed to test whether the use of sCD59 will save costs for health care systems by improving the patient's health outcomes.

To conclude, plasma and PDF levels of sCD59 could identify patients prone to progressive loss of membrane function, while plasma sCD59 might help to select patients at risk of loss of RRF. However, the underlying mechanisms of this observation are unknown and warrant further investigation. In addition, prospective studies in larger PD populations are needed to validate our results.

### Highlights

The European Training and Research in Peritoneal Dialysis Network identified unmet needs in the management of PD starting with “tools to identify patients who are at the highest risk and to guide personalized interventions to improve the individual clinical outcomes of PD.”. Our findings indicate that soluble CD59 (sCD59) could be useful as part of a biomarker panel for stratification of PD patients at risk of developing membrane failure and loss of residual renal function. Accordingly, the identification of these patients, who are at the highest risk for PD-related complications, paves the way towards better prevention and early intervention to improve the clinical outcome in these individuals.

## Supplementary Information

Below is the link to the electronic supplementary material.

Supplementary file1 (DOCX 51 KB)

## Abbreviations

BMI: Body mass index
CD46: Membrane cofactor protein
CD55: Decay accelerating factor
CD59: Membrane attack complex-inhibitory protein
CKD: Chronic kidney disease
Cregs: Complement regulatory proteins
D/P: Dialysate-to-plasma concentration ratio
ESRD: End-stage renal disease
GPl: Glycophosphoinositol
HD: Hemodialysis
IQR: Interquartile range
PD: Peritoneal dialysis
PDF: Peritoneal dialysis fluid
PET: Peritoneal equilibration test
PMF: Peritoneal membrane failure
RRF: Residual renal function
sC5b-9: Soluble membrane attack complex
sCD59: Soluble CD59
