# Supplementary material for: Soluble CD59 in peritoneal dialysis: a potential biomarker for peritoneal membrane function
Source: J Nephrol. 2020 Dec 11;34(3):801–10. doi: 10.1007/s40620-020-00934-7 (PMC8192357; doi:10.1007/s40620-020-00934-7)
Supplement: Supplementary file 1 — Supplementary file1 (DOCX 51 KB) [file 40620_2020_934_MOESM1_ESM.docx]

**Supplementary data**

**Table S1** **– determinants of peritoneal dialysate fluid levels of soluble CD59**

|  | **Univariate analysis** | |  |
| --- | --- | --- | --- |
|  | *St. Beta* | *P-value* |  |
| Age  *(years)* | − 0.015 | 0.92 |  |
| Sex  *(Female)* | − 0.125 | 0.40 |  |
| Dialysis vintage  *(months)* | − 0.001 | 0.99 |  |
| Residual renal function  *(mL/min/1.73m^2^)* | − 0.255 | 0.08 |  |
| Lean tissue index  *(kg/m^2^)* | − 0.100 | 0.54 |  |
| Fat tissue index  *(kg/m^2^)* | − 0.018 | 0.91 |  |
| Body mass index (BMI) *(kg/m^2^)* | − 0.034 | 0.82 |  |
| Overhydration  (*%*) | **0.389** | **0.014** |  |
| Mean arterial pressure (*mm/Hg*) | 0.074 | 0.63 |  |
| Baseline transport status *(D/P creatinine)* | 0.279 | 0.06 |  |
| Automated PD  *(%*) | − 0.261 | 0.07 |  |
| Diabetes  (*%*) | 0.086 | 0.56 |  |
| Protein loss  (*g/dL*) | − 0.180 | 0.29 |  |
| Plasma sCD59  *(ng/mL)* | 0.234 | 0.11 |  |
| PDF sC5b-9  *(ng/mL)* | 0.231 | 0.11 |  |

Univariate logistic regression analysis of peritoneal dialysis fluid (PDF) levels of soluble CD59 (sCD59) with clinical parameters was conducted. Next, multivariate logistic regression using the forward selection was performed with parameters that significantly associated (*P*-value<0.05) in univariate analysis, to identify independent determinants of PDF sCD59. Data are presented as standardized beta coefficient with corresponding *P*-value. Bold letters indicate a *P*-value < 0.05. Abbreviations: BMI, body mass index; PD, peritoneal dialysis; PDF, peritoneal dialysis fluid; sC5b-9, soluble C5b-9; sCD59, soluble CD59; D/P, Dialysate-to-plasma concentration ratio.

**Table S2** **– determinants of plasma levels of soluble CD59 in peritoneal dialysis**

|  |  | **Univariate analysis** | |  | **Multivariate analysis** | |
| --- | --- | --- | --- | --- | --- | --- |
|  |  | *St. Beta* | *P-value* |  | *St. Beta* | *P-value* |
| Age  *(years)* |  | − 0.034 | 0.82 |  |  |  |
| Sex  *(Female)* |  | 0.002 | 0.99 |  |  |  |
| Dialysis vintage  *(months)* |  | 0.239 | 0.12 |  |  |  |
| Residual renal function  *(mL/min/1.73m^2^)* |  | − **0.623** | **<0.001** |  | − **0.613** | **<0.001** |
| Lean tissue index  *(kg/m^2^)* |  | − 0.059 | 0.72 |  |  |  |
| Fat tissue index  *(kg/m^2^)* |  | − 0.098 | 0.55 |  |  |  |
| Body mass index (BMI) *(kg/m^2^)* |  | 0.075 | 0.63 |  |  |  |
| Overhydration  (*%*) |  | 0.194 | 0.24 |  |  |  |
| Mean arterial pressure (*mm/Hg*) |  | **0.418** | **0.005** |  | **0.319** | **0.009** |
| Baseline transport status *(D/P creatinine)* |  | − 0.084 | 0.58 |  |  |  |
| Automated PD  *(%*) |  | − 0.004 | 0.98 |  |  |  |
| Diabetes  (%) |  | − 0.036 | 0.81 |  |  |  |
| Protein loss  *(g/dL*) |  | − 0.256 | 0.13 |  |  |  |

Univariate logistic regression analysis of plasma soluble CD59 (sCD59) with clinical parameters was conducted. Next, multivariate logistic regression using the forward selection was performed with clinical parameters that significantly associated (*P*-value<0.05) in univariate analysis, to identify independent determinants of plasma sCD59. Data are presented as standardized beta coefficient with corresponding *P*-value. Bold letters indicate a *P*-value < 0.05. Abbreviations: BMI, body mass index; PD, peritoneal dialysis; sCD59, soluble CD59; D/P, Dialysate-to-plasma concentration ratio.

**Table S3 – determinants of transport status after 1 year.**

|  | **Univariate analysis** | |  | **Multivariate analysis** | |
| --- | --- | --- | --- | --- | --- |
|  | *St. Beta* | *P-value* |  | *St. Beta* | *P-value* |
| Age  *(years)* | − 0.114 | 0.52 |  |  |  |
| Sex  *(Female)* | 0.083 | 0.64 |  |  |  |
| PDF sCD59  *(ng/mL)* | **0.436** | **0.01** |  | 0.259 | 0.08 |
| Plasma sCD59  *(ng/mL)* | 0.016 | 0.93 |  |  |  |
| D/P-ratio of sCD59  *f* | 0.211 | 0.23 |  |  |  |
| PDF sC5b-9  *(ng/mL)* | 0.022 | 0.90 |  |  |  |
| Dialysis vintage  *(months)* | − **0.399** | **0.021** |  | − 0.148 | 0.35 |
| Residual renal function *(mL/min/1.73m^2^)* | − 0.066 | 0.71 |  |  |  |
| Lean tissue index  *(kg/m^2^)* | 0.058 | 0.76 |  |  |  |
| Fat tissue index  *(kg/m^2^)* | − 0.349 | 0.06 |  |  |  |
| Body mass index (BMI) *(kg/m^2^)* | − 0.336 | 0.06 |  |  |  |
| Overhydration  (*%*) | **0.434** | **0.019** |  | 0.062 | 0.75 |
| Mean arterial pressure (*mm/Hg*) | 0.204 | 0.26 |  |  |  |
| Baseline transport status  *(D/P creatinine)* | **0.649** | **<0.001** |  | **0.682** | **<0.001** |
| Protein loss  (*g/dL*) | − 0.070 | 0.72 |  |  |  |
| Diabetes  (*%*) | 0,214 | 0.23 |  |  |  |
| Automated PD  *(%*) | − 0.169 | 0.34 |  |  |  |

Univariate logistic regression analysis of transport status with clinical parameters was conducted. Transport status measured by dialysate-to-plasma concentration ratio (D/P) for creatinine using the modified peritoneal equilibration test (PET). Next, multivariate logistic regression using the forward selection was performed with parameters that significantly associated (*P*-value<0.05) in univariate analysis, to identify independent determinants of transport status. Data are presented as standardized beta coefficient with corresponding *P*-value. Bold letters indicate a *P*-value < 0.05. Abbreviations: BMI, body mass index; PD, peritoneal dialysis; PDF, peritoneal dialysis fluid; sC5b-9, soluble C5b-9; sCD59, soluble CD59; D/P, Dialysate-to-plasma concentration ratio.

**Table S4 – determinants of loss of diuresis during follow-up**

|  | **Univariate analysis** | |  | **Multivariate analysis** | |
| --- | --- | --- | --- | --- | --- |
|  | *St. Beta* | *P-value* |  | *St. Beta* | *P-value* |
| Age  *(years)* | 1.012 | 0.73 |  |  |  |
| Sex  *(Female)* | 1.869 | 0.45 |  |  |  |
| Plasma sCD59  *(ng/mL)* | **1.086*** | **0.003** |  | **1.116*** | **0.005** |
| PDF sCD59  *(ng/mL)* | 1.116* | 0.72 |  |  |  |
| DP-ratio of sCD59 | 0.297 | 0.33 |  |  |  |
| PDF sC5b-9  *(ng/mL)* | 1.007 | 0.52 |  |  |  |
| Dialysis vintage  *(months)* | 1.015 | 0.27 |  |  |  |
| Residual renal function *(mL/min/1.73m^2^)* | **0.530** | **0.02** |  | 0.726 | 0.41 |
| Lean tissue index  *(kg/m^2^)* | 0.926 | 0.56 |  |  |  |
| Fat tissue index  *(kg/m^2^)* | 0.849 | 0.09 |  |  |  |
| Body mass index (BMI) *(kg/m^2^)* | 0.977 | 0.63 |  |  |  |
| Overhydration  *(%)* | 1.030 | 0.48 |  |  |  |
| Mean arterial pressure (*mm/Hg*) | 1.032 | 0.29 |  |  |  |
| Baseline transport status  *(D/P creatinine)* | 1.406 | 0.95 |  |  |  |
| Protein loss  (*g/dL*) | **81.06** | **0.022** |  | **3873** | **0.004** |
| Diabetes  *(%)* | 0.040 | 0.55 |  |  |  |
| Automated PD  *(%*) | 0.045 | 0.69 |  |  |  |

During median follow-up of 14 months, 6 (12.5%) PD patients developed loss of diuresis, defined as an as a urinary output of less than 400mL per day. Univariate cox regression analysis of loss of diuresis with clinical parameters was conducted. Next, multivariate cox regression using the forward selection was performed with parameters that significantly associated (*P*-value<0.05) in univariate analysis, to identify independent determinants of loss of diuresis. Data are presented as standardized beta coefficient with corresponding *P*-value. Bold letters indicate a *P*-value < 0.05. * standardized beta coefficient per 10ng/mL increase in sCD59 levels. Abbreviations: BMI, body mass index; PD, peritoneal dialysis; PDF, peritoneal dialysis fluid; sC5b-9, soluble C5b-9; sCD59, soluble CD59; D/P, Dialysate-to-plasma concentration ratio.
